# Arbuscular Mycorrhizal Symbiosis: Plant Friend or Foe in the Fight Against Viruses?

**DOI:** 10.3389/fmicb.2019.01238

**Published:** 2019-06-04

**Authors:** Laura Miozzi, Anna Maria Vaira, Marco Catoni, Valentina Fiorilli, Gian Paolo Accotto, Luisa Lanfranco

**Affiliations:** ^1^Institute for Sustainable Plant Protection, National Research Council of Italy (IPSP-CNR), Turin, Italy; ^2^School of Biosciences, University of Birmingham, Birmingham, United Kingdom; ^3^Department of Life Sciences and Systems Biology, University of Turin, Turin, Italy

**Keywords:** arbuscular mycorrhiza, plant virus, mycorrhiza-induced resistance, plant-AMF-pathogen interaction, priming

## Abstract

Plant roots establish interactions with several beneficial soil microorganisms including arbuscular mycorrhizal fungi (AMF). In addition to promoting plant nutrition and growth, AMF colonization can prime systemic plant defense and enhance tolerance to a wide range of environmental stresses and below-ground pathogens. A protective effect of the AMF against above-ground pathogens has also been described in different plant species, but it seems to largely rely on the type of attacker. Viruses are obligate biotrophic pathogens able to infect a large number of plant species, causing massive losses in crop yield worldwide. Despite their economic importance, information on the effect of the AM symbiosis on viral infection is limited and not conclusive. However, several experimental evidences, obtained under controlled conditions, show that AMF colonization may enhance viral infection, affecting susceptibility, symptomatology and viral replication, possibly related to the improved nutritional status and to the delayed induction of pathogenesis-related proteins in the mycorrhizal plants. In this review, we give an overview of the impact of the AMF colonization on plant infection by pathogenic viruses and summarize the current knowledge of the underlying mechanisms. For the cases where AMF colonization increases the susceptibility of plants to viruses, the term “mycorrhiza-induced susceptibility” (MIS) is proposed.

## Introduction

### Effect of Mycorrhizal Colonization on Plant Responses to Biotic Stress

In natural environments, plants interact with pathogenic and beneficial microorganisms that might affect their growth, performance and survival. Arbuscular mycorrhizal fungi (AMF) (subphylum Glomeromycotina) (Spatafora et al., 2016) establish a mutualistic association with c. 85% of land plants, providing substantial benefits to plant growth and fitness (Jung et al., 2012; Auge et al., 2015). As a consequence of the improved mineral nutrition, AMF colonized plants often display increased biomass and productivity (Bona et al., 2016; Fiorilli et al., 2018). AMF root colonization induces a systemic effect also evident on epigeous portions of the plant (Fiorilli et al., 2009; Zouari et al., 2014) and exerts beneficial impacts beyond the nutritional status improvement, i.e., an enhanced ability to cope with biotic and abiotic stresses. This advantage relies on physiological and metabolic changes that take place in the plant upon AMF colonization (Fritz et al., 2006; Auge et al., 2015; Fiorilli et al., 2018) and proposes AM symbiosis as a biocontrol agent, impacting on the outcome of below- and above-ground interactions with other organisms.

Enhanced resistance of mycorrhizal plants against soilborne pathogens was often observed (Whipps, 2004), while contrasting results have been obtained for above-ground attackers (Pozo and Azcón-Aguilar, 2007). In roots, the bio-protective effect exerted by AMF seems to rely on several biotic factors such as fungal/host genotypes, mycorrhization degree and soil microbiota alteration, including development of pathogen antagonism and accumulation of defensive compounds (Pozo et al., 2002; Vierheilig et al., 2008; Cameron et al., 2013). The effects on above-ground pathogens seems to greatly depend on the pathogen lifestyle (Shaul et al., 1999; Fiorilli et al., 2011; Miozzi et al., 2011; Campos-Soriano et al., 2012; Jung et al., 2012; Song et al., 2013, 2015; Sanchez-Bel et al., 2016).

The boost of basal defenses in mycorrhizal plants was defined mycorrhiza-induced resistance (MIR) and several studies pointed to priming (Martinez-Medina et al., 2016) as a main mechanism operating in MIR (Pozo and Azcón-Aguilar, 2007; Cameron et al., 2013). Cameron et al. (2013) proposed that MIR is a cumulative effect of plant responses to mycorrhizal colonization, able to confer protection against a wide range of challengers, including biotrophic and necrotrophic pathogens, nematodes and insects. MIR, at least in shoots, seems to be a two-step process with a preliminary induction of a broad range of defense genes (including chitinases, glucanases and Pathogenesis Related (PR) proteins) (Spanu et al., 1989; Liu et al., 2007; Fiorilli et al., 2009) during AMF colonization, followed by a faster and stronger activation of pathogen-specific defense genes upon pathogen challenge (Campos-Soriano et al., 2012; Fiorilli et al., 2018). The main actors proposed to be involved in this process are plant hormones, i.e., salicylic acid (SA), jasmonic acid (JA) and its derivates oxylipins, ethylene and probably abscisic acid (ABA), whose level changes during the different steps of mycorrhizal symbiosis (Foo et al., 2013; Pozo et al., 2015; see section “Conclusion and Perspectives”). It is tempting to speculate that, beside the genetic, molecular and physiological mechanisms, other factors could affect MIR such as AMF associated endobacteria and virome (Bonfante and Desirò, 2017; Turina et al., 2018). Pioneering studies indicate that AMF endobacteria may improve the fungal ecological fitness (Salvioli et al., 2016) and promote antioxidative responses in both fungal and plant hosts (Vannini et al., 2016). However, data on the impact of endobacteria and mycoviruses (Ezawa et al., 2015) on AMF phenotypic expression and higher order biological interactions are scarce and deserve further investigations.

### Virus Infection, Damage in Agriculture and Management at Various Scales

Viruses are obligate pathogens able to infect virtually all organisms, including plants. Their infection process depends on the host machinery, allowing the virus to multiply and spread in the host. In plants, virus infections generally induce a disease syndrome, with symptoms such as developmental abnormalities, necrosis and chlorosis. For all major agronomical crops, viral diseases cause huge losses in production and quality, representing a serious threat to global food security (Varma and Malathi, 2003). Virus infection has been often associated to a general reduction of plant performance, i.e., inhibition of photosynthesis (Rahoutei et al., 2000), decrease of biomass (van Mölken and Stuefer, 2011) and pollen production (Harth et al., 2016), although recent evidences suggest that virus infection may improve drought tolerance (Xu et al., 2008; Pantaleo et al., 2016). Majority of viruses spread among plants very efficiently exploiting as vectors other organisms (mostly insects) characterized by a high level of mobility. Since climate changes can favor insect colonization of new habitats (Pureswaran et al., 2018), many viral diseases are representing an emerging problem in agriculture (Varma and Malathi, 2003; Rojas and Gilbertson, 2008; Ghini et al., 2011).

Sustainable and effective approaches to limit viral diseases include the development of viral-resistant/tolerant crop, the integration of crop management strategies to reduce the disease spreading (Nicaise, 2014), the introgression of resistance genes (e.g., NBS-LRR) from wild accessions, the use of transgenic plants expressing viral components, able to interfere with viral infection mechanisms at RNA or protein level. Unfortunately, these strategies are not immediately applicable to uncharacterized emerging viral pathogens.

AMF inoculation has been proposed as a cost-effective and sustainable solution for plant virus control. However, despite some information were available from the early 70’s, the studies on the effects of AMF on plant-virus interactions are surprisingly low and contradictory. The aim of this review is to summarize the actual knowledge on the effect of AMF on virus infection and the underlying mechanisms. We propose the term “mycorrhiza-induced susceptibility” (MIS) for the cases where a better performance of the virus, defined by replication efficiency and induced symptomatology, is observed in mycorrhizal plants. Furthermore, the state of the art on the effect of virus infection on mycorrhization will be reported; finally, we suggest different aspects that deserve further investigations.

## Plant Protective Effect of the Amf Colonization Against Viral Infection

Up to now, three studies highlighted a plant protective effect of the AMF colonization against viral infection. All of them considered *Solanaceae* or *Cucurbitaceae* plant species and positive single stranded RNA viruses, with the exception of Maffei et al. (2014) that focused on a single-stranded circular DNA geminivirus (**Table 1**). In Maffei et al. (2014), previously AMF-colonized tomato plants displayed attenuated symptoms and reduced virus titre when infected by *Tomato yellow leaf curl Sardinia virus* (TYLCSV) although AMF colonization could not contrast the reduction of root biomass induced by the virus. Since, TYLCSV encodes proteins able to interact with the plant hormone pathways (Lozano-Durán et al., 2011) and particularly with JA, a key hormone in MIR (Cameron et al., 2013), the authors hypothesized that the high JA level in mycorrhizal plants creates an unfavorable environment for TYLCSV, limiting its replication and reducing symptoms severity. This hypothesis is in agreement with the priming effect induced by JA exogenous application during geminivirus infection, which was sufficient to reduce symptoms and viral titre in *Beet curly top virus*-infected plants (Lozano-Durán et al., 2011).

**Table 1:** AMF-plant-virus biological systems investigated; in the upper section are listed the case studies reporting a protective effect of AMF against viral infection while in the lower section are listed those reporting a detrimental effect.

Plant (family)	Fungus	Virus (genus, family)	Virus type (baltimore classification)	Effect of AMF on virus infection	Plant tissues considered	Effect of virus infection on AMF-colonized plant	References
Tomato (Solanaceae)	*Funneliformis mosseae* (formerly *Glomus mosseae*)	*Tomato yellow leaf curl Sardinia virus* (*Begomovirus*, *geminiviridae*)	ssDNA (Group II)	Decreased virus titre, milder symptoms	Leaves, roots	Reduction of roots fresh weight	Maffei et al., 2014
Potato (Solanaceae)	*Rhizophagus irregularis* (formerly *Glomus intraradices*)	*Potato virus Y* (*Potyvirus*, *potyviridae*)	Positive ssRNA (Gruppo IV)	Milder symptoms	Leaves, stems, roots	Increase of leaves and stems dry weight	Thiem et al., 2014
Tobacco (Solanaceae)	*Rhizophagus irregularis* (formerly *Glomus intraradices*)	*Tobacco mosaic virus* (*Tobamovirus*, *virgaviridae*)	Positive ssRNA (Gruppo IV)	No symptoms, decreased virus titre	Leaves	Not reported	Stolyarchuk et al., 2009
Cucumber (Cucurbitaceae)	*Rhizophagus irregularis* (formerly *Glomus intraradices*)	*Cucumber green mottle mosaic* virus (*Tobamovirus*, *virgaviridae*)	Positive ssRNA (Gruppo IV)	Milder symptoms, reduced virus titre	Leaves	Not reported	Stolyarchuk et al., 2009
Tomato (Solanaceae)	*Funneliformis macrocarpa* (formerly *Endogone macrocarpa*)	*Tomato aucuba mosaic virus*^∗^ (*Tobamovirus*, *Virgaviridae*)	Positive ssRNA (Gruppo IV)	Increased virus titre	Leaves, roots	Not reported	Daft and Okusanya, 1973
Tomato (Solanaceae)	*Funneliformis macrocarpa* (formerly *Endogone macrocarpa*)	*Potato virus X* (*Potexvirus*, *Alphaflexiviridae*)	Positive ssRNA (Gruppo IV)	Increased virus titre	Leaves, roots	Not reported	Daft and Okusanya, 1973
Petunia (Solanaceae)	*Funneliformis macrocarpa* (formerly *Endogone macrocarpa*)	*Arabis mosaic virus* (*Nepovirus*, *secoviridae*)	Positive ssRNA (Gruppo IV)	Increased virus titre	leaves, roots	Not reported	Daft and Okusanya, 1973
Strawberry (Rosaceae)	*Funneliformis macrocarpa* (formerly *Endogone macrocarpa*)	*Arabis mosaic virus* (*Nepovirus*, *secoviridae*)	Positive ssRNA (Gruppo IV)	Increased virus titre	Leaves, roots	Not reported	Daft and Okusanya, 1973
Tomato (Solanaceae)	*Glomus* sp.	*Tobacco mosaic virus* (*Tobamovirus*, *virgaviridae*)	Positive ssRNA (Gruppo IV)	Increased virus titre, more severe symptoms	Roots, whole plant (for symptoms evaluation)	Increase of roots fresh weight	Jabaji-Hare and Stobbs, 1984
Sour orange (Rutaceae)	*Claroideoglomus etunicatum* (formerly *Glomus etunicatum*)	*Citrus tristeza virus* (*Closterovirus, closteroviridae*)	Positive ssRNA (Gruppo IV)	No difference	whole plant	Reduction of roots fresh weight and plant growth	Nemec and Myhre, 1984
Tobacco (Solanaceae)	*Rhizophagus irregularis* (formerly *Glomus intraradices*)	*Tobacco mosaic virus* (*Tobamovirus*, *virgaviridae*)	Positive ssRNA (Gruppo IV)	more severe symptoms	leaves	Not reported	Shaul et al., 1999
Potato (Solanaceae)	*Rhizophagus irregularis* (formerly *Glomus intraradices*)	*Potato virus Y* (*Potyvirus*, *potyviridae*)	Positive ssRNA (Gruppo IV)	Increased virus titre, more severe symptoms	leaves,whole plant (for symptoms evaluation)	Reduction of shoots length, fresh and dry weight, and tuber weight. Slight reduction of chlorophyll content	Sipahioglu et al., 2009
Tomato (Solanaceae)	*Rhizophagus irregularis* (formerly *Glomus intraradices*)	*Tobacco mosaic virus* (tobamovirus, *virgaviridae*)	Positive ssRNA (Gruppo IV)	Increased virus titre	Leaves	Not reported	Stolyarchuk et al., 2009
Tomato (Solanaceae)	*Funneliformis mosseae* (formerly *Glomus mosseae*)	*Tomato spotted wilt virus* (*Orthotospovirus*, *tospoviridae*)	Negative ssRNA (Group V)	Increased virus titre, more severe symptoms (lower recovery)	leaves, roots	Reduction of fresh weight of epigean and hypogean parts	Miozzi et al., 2011
*Bromus hordeaceus* L. (Poaceae)	*Rhizophagus irregularis* (formerly *Glomus intraradices*)	*Barley yellow dwarf virus* (*Luteovirus*, *luteoviridae*)	Positive ssRNA (Gruppo IV)	Increased virus titre (only with elevated CO2 concentration)	leaves	Not reported	Rùa et al., 2013
*Bromus hordeaceus* L. (Poaceae)	*Rhizophagus irregularis* (formerly *Glomus intraradices*)	*Cereal yellow dwarf virus* (*Polerovirus*, *luteoviridae*)	Positive ssRNA (Gruppo IV)	Increased virus titre (only with elevated CO2 concentration)	leaves	Not reported	Rùa et al., 2013
*Avena fatua* L. (Poaceae)	*Rhizophagus irregularis* (formerly *Glomus intraradices*)	*Barley yellow dwarf virus* (*Luteovirus*, *luteoviridae*)	Positive ssRNA (Gruppo IV)	Increased virus titre (only with elevated CO2 concentration)	leaves	Not reported	Rùa et al., 2013
*Avena fatua* L. (Poaceae)	*Rhizophagus irregularis* (formerly *Glomus intraradices*)	*Cereal yellow dwarf virus* (*Polerovirus*, *luteoviridae*)	Positive ssRNA (Gruppo IV)	Increased virus titre (only with elevated CO2 concentration)	leaves	Not reported	Rùa et al., 2013


*a strain of tobacco mosaic virus.

Differently from Maffei et al. (2014), Thiem et al. (2014) investigated the effect of mycorrhizal colonization on potato plants already infected by *Potato virus Y* (PVY): milder symptoms and a significant stimulation of shoot growth were observed in PVY-infected plants inoculated with *Rhizophagus irregularis*.

Finally, tobacco and cucumber plants colonized by *R. irregularis* and infected by *Tobacco mosaic virus* (TMV) and *Cucumber green mottle mosaic virus* (CGMMV), respectively, showed reduced disease symptoms and virus titre if compared to non-mycorrhizal plants (Stolyarchuk et al., 2009); the same authors, in the TMV-tomato system, observed that the content of viral antigens in mycorrhizal plants in respect to non-mycorrhizal ones changed overtime, being equal at 14 days post viral inoculation (dpi), then increasing and subsequently decreasing from 21 to 49 dpi and 56 dpi, respectively.

## Negative Impact of Amf Colonization on Plant Response to Virus Infection

Several studies report increased virus multiplication and/or symptom severity in infected mycorrhizal plants. They considered plants belonging to the *Solanaceae*, *Rosaceae* and *Poaceae* families and mostly dealt with single stranded RNA viruses (**Table 1**). Results indicate that mycorrhizal colonization facilitates or enhances virus multiplication, suggesting a prevailing detrimental effect of AMF on plant virus infection, for which we propose the term “mycorrhiza-induced susceptibility” (MIS). Even if, in the first days after inoculation by *Tomato aucuba mosaic virus* (now a TMV strain), the virus titre in tomato plants colonized by *Funneliformis macrocarpa* (formerly *Endogone macrocarpa*) was lower in respect to the control ones, at 8–12 dpi, it became higher in mycorrhizal plants and increased over time (Daft and Okusanya, 1973). Similar results were obtained in leaves and roots of both tomato and strawberry plants inoculated with *Potato virus X* (PVX) (Daft and Okusanya, 1973). No data on plant biomass or performance were reported. These authors observed a similar virus titre increment in infected non-mycorrhizal plants grown with increased concentration of soluble phosphate, and suggested that enhanced viral multiplication could be a general consequence of increased phosphorus availability provided by the symbiosis. This hypothesis relies on the established correlation between phosphate nutrition and TMV infection in tobacco plants (Bawden and Kassanis, 1950; Kassanis, 1953) and was also suggested by Sipahioglu et al. (2009) to explain the increase in PVY titre and symptomatology in potato plants colonized by *R. irregularis*. It is interesting to note that the results of Sipahioglu et al. (2009) were in contrast with those of Thiem et al. (2014) even if the authors considered a similar biological system (potato, *R. irregularis*, PVY) and experimental design (mycorrhization of already PVY-infected plants). Sipahioglu et al. (2009), also observed a reduction in the length, fresh and dry weight of shoots, and in tubers weight, as well as a slight reduction in leaves chlorophyll content in virus-infected mycorrhizal plants.

The MIS outcome, consisting in viral titre increase and worsening of symptoms, was confirmed by Jabaji-Hare and Stobbs (1984) and Shaul et al. (1999) respectively in TMV-infected tomato and tobacco plants colonized by *Glomus* sp. Jabaji-Hare and Stobbs (1984) also observed an increase of roots fresh weight in the virus-infected mycorrhizal plants when compared to non-infected mycorrhizal ones. The results of Shaul et al. (1999) suggest that MIS is not entirely dependent by the improved plant nutritional status; his study excluded any effect of improved phosphorus nutrition and linked the increased plant susceptibility to viral infection with the delay in PR proteins induction in mycorrhizal plants.

More recently, Miozzi et al. (2011) observed increased *Tomato spotted wilt virus* (TSWV) titre in infected mycorrhizal tomato plants at 34 and 56 dpi, but not at 14 dpi, compared to non-mycorrhizal controls. A delay in recovery (symptoms disappearance/reduction in plants initially showing severe disease; Pennazio, 2010) was observed in TSWV-infected mycorrhizal plants at 34 dpi, but disappeared later (56 dpi). Similarly to Shaul et al. (1999), these authors observed a reduction in the number and fold-change of PR proteins coding genes in TSWV-infected mycorrhizal plants when compared with TSWV-infected non-mycorrhizal plants. Since the MIR-related JA-dependent defense priming is hypothesized to be linked to the partial suppression of the salicylic acid (SA)-dependent response (Pozo and Azcón-Aguilar, 2007), it was proposed that the SA level increase induced by TSWV infection may prevent the MIR-mediated response. Within the TSWV-AMF-plant interaction an involvement of ABA and a more complex cross-talk among phytohormones, not limited to SA and JA, were also postulated (Miozzi et al., 2011). Indeed, the pretreatment with ABA can suppress the non-pathogenesis related protein 1 (NPR1) gene, an important regulatory component of SA signaling involved in PR genes activation (Dong, 2004; Yasuda et al., 2008). The long-term changes in virus titre and symptomatology observed by Miozzi et al. (2011) are in agreement with the observation that the protective or detrimental effect of mycorrhizal colonization on viral infection can substantially change over time (Daft and Okusanya, 1973; Stolyarchuk et al., 2009) underling that timing is a key parameter in the complex interaction among plant, viruses and AMF. In agreement with other studies here reported, Miozzi and co-authors (2011) observed that the AMF colonization failed to compensate the biomass reduction induced by the virus (**Table 1**).

Rùa et al. (2013), addressing the possible consequences of climate changes and increase of atmospheric CO_2_, observed that, under elevated CO_2_ concentration, the titre of both *Barley yellow dwarf virus* (BYDV) and *Cereal yellow dwarf virus* (CYDV) increases in the grasses *Bromus hordeaceus* L. and *Avena fatua* L. colonized by *R. irregularis*; no differences were observed under normal CO_2_ concentration.

Less investigated is the effect of mycorrhization on fruit trees. Nemec and Myhre (1984), studying the changes induced by *Claroideoglomus etunicatum* (formerly *Glomus etunicatum*) on sour orange and Duncan grapefruit seedlings infected by *Citrus tristeza virus* and *Citrus leaf rugose virus*, respectively, observed that AMF colonization did not significantly reduce the pathogenic effect caused by virus infection. A reduction of roots fresh weight and plant growth in the virus-infected mycorrhizal plants in respect to non-infected mycorrhizal plants was found. Similarly to Thiem et al. (2014) and Sipahioglu et al. (2009), in this work, plants were inoculated with AMF after virus infection.

## The Impact of Viral Infection on Mycorrhization

Most studies addressing the plants-viruses-AMF interaction mainly focused on the effect of AMF on virus infection; however, viral infection can impact mycorrhization. In this regard, Nemec and Myhre (1984) observed that the number of fungal spores and the percentage of mycorrhization were generally higher in not-infected plants in respect to infected ones. Maffei et al. (2014) observed that the frequency of mycorrhization moderately but significantly increased in TYLCSV-infected mycorrhizal plants compared to not-infected mycorrhizal ones. However, no differences were observed in intensity of mycorrhization, abundance of arbuscules within colonized areas and percentage of the root system with arbuscules, suggesting that the onset and spread of TYLCSV throughout the whole plant may not significantly interfere with the *F. mosseae* intraradical development. This result is consistent with the up-regulation, in both mycorrhizal and TYLCSV-infected mycorrhizal plants, of five selected plant genes previously described as mycorrhiza-responsive and preferentially expressed in arbuscule-containing cells. Similarly, Sipahioglu et al. (2009) reported equal degree of mycorrhizal colonization in PVY-infected and healthy mycorrhizal potato plants. Finally, Rùa et al. (2013) observed that, only under elevated CO_2_ concentration, BYDV and CYDV infection increased the fungal colonization of roots, suggesting that, in this case and condition, AMF and virus interacted to stimulate each other success.

## Conclusion and Perspectives

**FIGURE 1:**
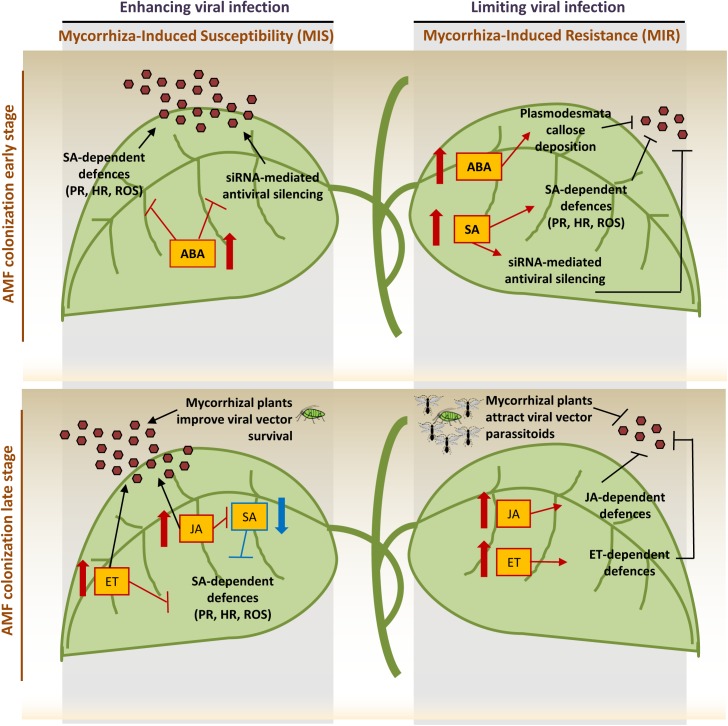
Visual dissection* of the events during AMF colonization and virus infection: changes in hormones levels and related processes may enhance (left) or limit (right) viral infection leading to the final outcome of the complex tripartite interaction. In the early stage of mycorrhization, the increase of salicylic acid (SA) induces the priming of SA-dependent defenses, the major defensive pathway against viruses, and enhances the siRNA-mediated antiviral silencing. At the same time, abscisic acid (ABA) increases with both positive and negative consequences on plant defenses: it induces callose deposition on plasmodesmata, limiting cell-to-cell movement, suppresses SA signaling transduction, thus inhibiting defenses controlled by this pathway and weakens siRNA-antiviral system. In the late AMF colonization stage, the increase of jasmonate (JA) and ethylene (ET) induces the priming of JA- and ET-dependent defenses. JA has been shown to reduce viral symptoms at early infection stage but increase susceptibility in late infection stage; on the other hand, JA treatment decreases viral titre during geminivirus infection. ET antagonizes the pathway downstream the SA signaling and may be involved in symptom development, viral systemic movement and formation of necrotic lesions. However, spraying plants with the ET precursor 1-aminocyclopropane-l-carboxylic acid may reduce viral titre. Finally, mycorrhizal plants have been shown to improve aphid survival and increase attractiveness toward aphids parasitoids. PR: pathogenesis-related proteins, HR: hypersensitive response, ROS: reactive oxygen species, brown hexagones indicate viral particles (Lozano-Durán et al., 2011; Cameron et al., 2013; Alazem and Lin, 2015; Volpe et al., 2018).

The picture arising from the reported studies, even if very complex, suggests a prevailing detrimental effect of AMF on plant virus infection, for which we propose the term “mycorrhiza-induced susceptibility” (MIS). Indeed, the interaction among virus, AMF and plant is a complex system where several factors, including viral pathogen lifestyle, plant nutritional status and timing of interaction, can move the dynamic equilibrium toward the final establishment of a MIS or MIR outcome. A key role is probably played by the hormonal crosstalk that finely tunes both plant-AMF and plant-virus interactions (**Figure 1**). In the early stage of AMF colonization, MIR has been associated with SAR (Systemic Acquired Resistance)-like priming of SA-dependent genes, while in the later stage, MIR coincides with priming of JA- and ethylene-dependent defenses. In addition, ABA has been proposed as a new candidate acting as a complementary long-distance signal controlling MIR, and several reports considered JA and its derivates (i.e., oxylipins) as key signals operating in this process (Cordier et al., 1998; Van Wees et al., 2008; Van der Ent et al., 2009; Jung et al., 2012; Song et al., 2013, 2015). In parallel, SA-dependent defenses are the major defensive pathway against viruses while ABA may act either positively and negatively on plant defenses against viruses, respectively, limiting cell-to-cell movement by inducing callose deposition on plasmodesmata, or inhibiting SA-mediated defenses (Alazem and Lin, 2015). Interestingly, SA and ABA may also interact with the plant RNA silencing machinery, respectively inducing dicer-like 1 and 2 and RNA-dependent-RNA polymerase 1 and 2 in virus infected plants (Campos et al., 2015) and regulating the expression of argonaute genes involved in plant antiviral defense (Várallyay et al., 2010; Harvey et al., 2011; Alazem and Lin, 2015). This mechanism could interfere with the siRNA-mediated antiviral plant defense (Alazem and Lin, 2015) and adding further level of complexity to the mechanisms regulating the plant-virus-AMF interaction. Indeed, in this context, the role of siRNAs/miRNAs has not been explored so far, but may be a key element in determining the final interaction outcome. Beyond plant siRNAs, fungal siRNAs have been recently proposed having a functional significance in the *trans*-kingdom communication between the AMF and its host plant (Silvestri et al., 2019), suggesting that their possible role in the final outcome of viral infection in mycorrhizal plants should be addressed.

The complexity of the AMF-plant-virus interaction may further increase if considering that mycorrhiza can impact other trophic levels such as the interaction between plants and insects (Gehring and Bennett, 2009), including those acting as viral vectors. Indeed, AMF *R. irregularis* can improve the survival of the aphid *Macrosiphum euphorbiae*, vector of *Cucumber mosaic virus*, thus possibly improving viral spread, but also activate indirect defenses, attracting the aphid parasitoid *Aphidius ervi* (Volpe et al., 2018).

These observations highlight the intricate network of processes that regulate the plant-virus-AMF interaction, and, far to be conclusive, indicate that several factors able to direct the dynamic equilibrium of the system toward a MIS or MIR outcome remain to be evaluated such as the different changes induced by AMF colonization performed before or after viral infection, and the importance of timing in evaluating the interaction outcome. Moreover, since only few AMF have been analyzed so far, future studies should consider different AMF species and isolates (Sikes, 2010; Turrini et al., 2018) and even AMF consortia for detecting possible synergistic/antagonistic effects. Finally, it must be emphasized that, based on current knowledge, drawing conclusions on the efficacy of AMF to act as biocontrol agents in agricultural environments is extremely difficult, since, in these conditions, many other biotic and abiotic factors have the potential to interfere with the final MIS or MIR outcome.

## Author Contributions

All authors listed have made a substantial, direct and intellectual contribution to the work, and approved it for publication.

## Conflict of Interest Statement

The authors declare that the research was conducted in the absence of any commercial or financial relationships that could be construed as a potential conflict of interest.
